# The effects of care bundles on patient outcomes: a systematic review and meta-analysis

**DOI:** 10.1186/s13012-017-0670-0

**Published:** 2017-11-29

**Authors:** Jacqueline F. Lavallée, Trish A. Gray, Jo Dumville, Wanda Russell, Nicky Cullum

**Affiliations:** 1 Division of Nursing, Midwifery and Social Work, University of Manchester, Manchester Academic Health Science Centre, Room 3.331, Jean McFarlane Building, Oxford Road, Manchester, M13 9PL England; 2 Collaboration for Leadership in Applied Health Research and Care (CLAHRC) Greater Manchester, Manchester, England; 3 Manchester Centre for Health Psychology, University of Manchester, Manchester, England; 4 Primary Care & Research Services, Keele University, Newcastle-under-Lyme, England; 5 Research and Innovation Division, Manchester University NHS Foundation Trust, Manchester Academic Health Science Centre, Manchester, England

**Keywords:** Care bundle, Effectiveness, Implementation fidelity, Behaviour change

## Abstract

**Background:**

Care bundles are a set of three to five evidence-informed practices performed collectively and reliably to improve the quality of care. Care bundles are used widely across healthcare settings with the aim of preventing and managing different health conditions. This is the first systematic review designed to determine the effects of care bundles on patient outcomes and the behaviour of healthcare workers in relation to fidelity with care bundles.

**Methods:**

This systematic review is reported in line with the PRISMA statement for reporting systematic reviews and meta-analyses. A total of 5796 abstracts were retrieved through a systematic search for articles published between January 1, 2001, to February 4, 2017, in the Cochrane Central Register for Controlled Trials, MEDLINE, EMBASE, British Nursing Index, CINAHL, PsychInfo, British Library, Conference Proceeding Citation Index, OpenGrey trials (including cluster-randomised trials) and non-randomised studies (comprising controlled before-after studies, interrupted time series, cohort studies) of care bundles for any health condition and any healthcare settings were considered. Following the removal of duplicated studies, two reviewers independently screen 3134 records. Three authors performed data extraction independently. We compared the care bundles with usual care to evaluate the effects of care bundles on the risk of negative patient outcomes. Random-effect models were used to further explore the effects of subgroups.

**Results:**

In total, 37 studies (6 randomised trials, 31 controlled before-after studies) were eligible for inclusion. The effect of care bundles on patient outcomes is uncertain. For randomised trial data, the pooled relative risk of negative effects between care bundle and control groups was 0.97 [95% CI 0.71 to 1.34; 2049 participants]. The relative risk of negative patient outcomes from controlled before-after studies favoured the care bundle treated groups (0.66 [95% CI 0.59 to 0.75; 119,178 participants]). However, using GRADE, we assessed the certainty of all of the evidence to be very low (downgraded for risk of bias, inconsistency, indirectness).

**Conclusions:**

Very low quality evidence from controlled before-after studies suggests that care bundles may reduce the risk of negative outcomes when compared with usual care. By contrast, the better quality evidence from six randomised trials is more uncertain.

**Trial registration:**

PROSPERO, CRD42016033175

**Electronic supplementary material:**

The online version of this article (10.1186/s13012-017-0670-0) contains supplementary material, which is available to authorized users.

## Introduction

One of the main purposes of health research is to optimise health and healthcare by identifying effective healthcare interventions. Nevertheless, health research will only improve patient outcomes if the findings of research can be implemented into practice (where this is warranted) [1–3]. The translation of research findings into practice is a slow process [4]. Thus, a goal of implementation research is to improve patient outcomes by identifying the effective ways of translating research findings into practice [1]. Improving the quality of care and increasing research-informed practice has received much interest over the past decade [5–8]. Research-informed practice requires healthcare professionals to work and think differently [9] because providing the evidence is necessary but alone is not sufficient [10, 11]. So, more recently, research has looked at how we might change the behaviour of healthcare workers to facilitate the uptake of research-informed practice in healthcare [12–14].

To improve the quality of care and reduce the variations in care within intensive care units (ICUs), the Institute for Healthcare Improvement introduced the notion of care bundles [15, 16]. Care bundles contain three to five evidence-informed practices, which need to be delivered collectively and consistently with the aim of improving patient outcomes [16]. The Institute for Healthcare Improvement recommends that fidelity with care bundles should be at least 95% and every eligible patient should receive all of the elements included within the care bundle unless medically contraindicated [16]. Care bundles are used within healthcare for many different conditions (e.g. to prevent: ventilator-associated pneumonia, pressure ulcers). Whilst the elements of care within the care bundles formalise care, their success will be influenced by the implementation processes used to support the care bundle use in practice (e.g. shaping of knowledge, monitoring and feedback) [17]. Consequently, the behaviours of healthcare workers need to be targeted as part of the intervention [18].

Interventions aimed at changing health behaviours are often complex and comprise several components which have a synergistic effect [19]. Thus, care bundles are sometimes regarded as ‘complex interventions’ due to the number of components and their interaction within the care bundle; the context within which the care bundle is implemented; the number and variability of outcomes; the extent to which the care bundle can be tailored and the difficulty of performing the care bundle tasks. The Medical Research Council’s framework for developing and evaluating complex interventions recommends grounding complex interventions in theory to increase the likelihood of effectiveness [19]. Capitalising on behaviour change theory is important as the factors which influence the target behaviour; the active components of the intervention and the delivery of the intervention can be identified and selected [13].

Behaviour change techniques are the observable and replicable components of behaviour change interventions, often referred to as the ‘active ingredients’ [20–22]. Previous studies reporting the use of behaviour change interventions have employed a number of different behaviour change techniques, but they have been defined differently or unclearly which limits the evaluation and replication of these interventions [23]. To address this issue, a taxonomy of 93 behaviour change techniques [22] was developed and can be used to identify intervention components, enabling the standardisation of terms as well as the comparison of behaviour change techniques across studies. *Feedback on outcomes of behaviour, prompts/cues* and *instruction on how to perform a behaviour* are examples of behaviour change techniques commonly used to facilitate a behaviour change in healthcare workers [17, 24]. Identifying the specific behaviour change techniques employed during the implementation of care bundles could enable researchers and healthcare workers to understand which components were key when the implementation of a care bundle was successful. Moreover, by using standardised behaviour change language, comparisons with other care bundles in implementation research will be possible. Such standardised language and comparisons will increase our knowledge of the most suitable methods for implementing care bundles and facilitate the prediction and explanation of any subsequent behaviour change [25].

To date, systematic reviews of care bundles have been condition [26–35] or setting-specific [36–38]. Very few systematic reviews have explored the common behaviour change techniques employed to facilitate the implementation of care bundles and it is unknown which factors may impact on the success of care bundles. Therefore, the objectives of this review were to evaluate the effects of care bundles as tools for reducing the number of negative patient outcomes, to identify potentially effective approaches to the implementation of care bundles and to explore whether there are plausible factors that modify the effects of care bundles (e.g. healthcare settings, fidelity with the bundle, the number of care bundle elements, different implementation techniques).

## Methods

To maximise clarity and transparency, we have reported our review in line with the PRISMA statement for the reporting of systematic reviews and the meta-analyses of studies evaluating healthcare interventions [39].

### Eligibility criteria

We applied the following eligibility criteria:

#### Study design

Randomised trials (including cluster-randomised trials) and non-randomised trials (comprising controlled before-after studies, interrupted time series studies and cohort studies) were eligible for inclusion. Additionally, interrupted time series studies were required to have at least three data points both before and after the intervention. Conference abstracts were eligible if we were able to contact the authors and they provided sufficient information to allow a decision to be made regarding inclusion based on our eligibility criteria. We were only able to include English language articles due to resource constraints.

#### Participants

Evaluations of the impact of care bundles on patients of any age, in any setting and with any condition were eligible for inclusion.

#### Intervention

Studies were eligible for inclusion if they evaluated a care bundle. Our operational definition of a care bundle was informed by the Institute for Healthcare Improvement:

‘a small, straightforward set of evidence-based practices—generally three to five—that, when performed collectively and reliably, have been proven to improve patient outcomes’ [16].

#### Outcome measures

Primary outcome measures for the review were the number of negative patient outcomes (e.g. the number of central line-associated bloodstream infections per 1000 catheter days; mortality) and implementation fidelity (i.e. adherence with the care bundle).

Where study eligibility was in doubt due to a lack of information in the publication (e.g. conference abstracts), we attempted to contact the authors.

### Search strategy

The search strategy was developed using the terms based on intervention and outcomes (see Additional file 1 for search terms). To maximise retrieval, we searched the following databases: British Nursing Index (*n* = 811), CINAHL (*n* = 290), MEDLINE (*n* = 985), EMBASE (*n* = 986), CENTRAL (*n* = 1623), PsycINFO (*n* = 1101), British Library (*n* = 0), Conference Proceedings Citation Index (*n* = 0) and OpenGrey (*n* = 0). As the Institute for Healthcare Improvement developed the notion of care bundles in 2001, searches were restricted to the studies conducted during or after 2001 until 4 February 2017. The reference lists of the included articles were also checked.

### Data collection and analysis

#### Selection of studies

References were managed in EndNote (Clarivate Analytics, Philadelphia), which assisted with the identification and removal of duplicate studies, and imported into Covidence Systematic Review Software (Melbourne). Two reviewers (JL, WR), who were not blinded to study authors, screened the titles and abstracts before conducting a full-text review of the remaining studies. Where discrepancies occurred, we reached agreement through discussion.

#### Data extraction

Three reviewers (JL, WR, TG) independently performed data extraction using a pre-defined extraction sheet. We extracted the following data:

- Title
- Aims/objectives
- Study design
- Country of study
- Patient population (inclusion and exclusion criteria, age, co-morbidities, sex)
- Healthcare settings
- Care bundle content: the number and nature of the care bundle elements; the characteristics of those delivering and receiving the care bundle; the frequency with which the components were delivered and for how long
- Intervention content: we considered a care bundle to have been informed by theory if the authors explicitly stated using a relevant theory when describing either the development or implementation of the care bundle.
- Behaviour change techniques: a post hoc approach was taken where we retrospectively assigned the reported implementation techniques (e.g. training session) to one of the 93 behaviour change techniques according to the Behaviour Change Technique Taxonomy Version 1 [22]. Where several behaviour change techniques within the same category were used, this was counted as one (e.g. if ‘*monitoring of behaviour*’ and ‘*feedback on behaviour’* were used, according to the taxonomy, these would be classed as ‘*feedback and monitoring*’).
- Fidelity data relating to adherence to the care bundles were extracted from the data provided in the papers.
- Duration of follow-up
- Outcome measures
- Outcome data
- Funding source

#### Risk of bias assessment

Three reviewers (JL, WR, TG) independently assessed the included studies for their risk of bias, and we resolved disagreements through discussion. Interrater reliability was calculated using Cohen’s kappa [40]. The Cochrane Collaboration tool for assessing risk of bias [41] was used for randomised trials. The Cochrane Risk of Bias Assessment Tool: for Non-Randomised Studies of Interventions [42] was used to assess the risk of bias for non-randomised studies. We assessed the inter-rater reliability for the risk of bias judgements of the randomised trials (*K* = 0.82) and non-randomised studies (*K* = 0.70).

#### Measures of treatment effect

For dichotomous outcomes (e.g. negative patient outcomes), we calculated the risk ratio (RR) with 95% confidence intervals (CIs). A RR value of < 1 favoured the use of the care bundle (i.e. indicated a lower risk of the negative events with care bundles) and a value > 1 indicated more favourable outcomes when usual care was applied (i.e. there was a higher risk of the negative events with care bundles). Fidelity with the care bundle was recorded as a percentage indicating the extent to which the patient received either a particular care bundle element or the whole care bundle. For continuous data using the same scale, we used the difference in means (MD) with 95% CIs, and when different scales were used, we calculated the standardised difference in means (SMD) with 95% CIs.

#### Assessment of heterogeneity

We considered clinical and methodological heterogeneity: that is how participants, outcomes and characteristics (e.g. number of care bundle elements) varied between studies. This assessment was complemented by an assessment of statistical heterogeneity using the chi-squared test (statistically significant heterogeneity was indicated by a significance level of *P* < 0.1). In addition, *I* ^2^ [43] was also calculated, which is the percentage of total variation across studies due to heterogeneity. We followed the rubric that an *I* ^2^ of 0–40% indicates low heterogeneity [43] whilst 75 to 100% indicates very high heterogeneity [44]. A fixed effect analysis was planned when minimal clinical heterogeneity was supported by 0% statistical heterogeneity [45]. In cases where statistical heterogeneity was greater than 0%, we planned to use a random effects model.

#### Assessment of reporting biases

We planned to present funnel plots for meta-analyses comprising of 10 randomised trials or more to detect possible publication bias [41].

#### Dealing with missing data

We conducted a complete case analysis and dealt with missing data issues in the risk of bias assessment.

#### Data synthesis

A narrative summary of the characteristics of the included studies and a forest plot of study findings are presented. We planned to pool data across studies where possible but we anticipated high levels of clinical and methodological heterogeneity due to the broad review question. Thus, we planned to explore the heterogeneity by conducting considered subgroup analyses using Comprehensive Meta-Analysis software [46]. We pre-specified that the following study features may potentially explain some of the heterogeneity: study design, health condition, healthcare setting, the number of care bundle elements, the number of behaviour change techniques and the levels of fidelity with the care bundles. The first author (JL) undertook the data analysis and synthesis, and it was validated by the third author (JD). We pooled the data from each of the subgroups using a random-effect model and reported the principle measures of effect using 95% confidence intervals with risk ratios. As we present data from a random effect model, the reported results are the average effect for each subgroup. We used this approach to explore whether there was an underlying effect of care bundles and to guide future research [47, 48].

#### Sensitivity analysis

We planned to perform sensitivity analyses to explore the effect of the risk of bias by conducting a meta-analysis both with and without the studies assessed as being at a high or unclear risk of bias. However, this was not possible due to the limited number of studies assessed as being at a low risk of bias.

## Results

**Fig. 1:**
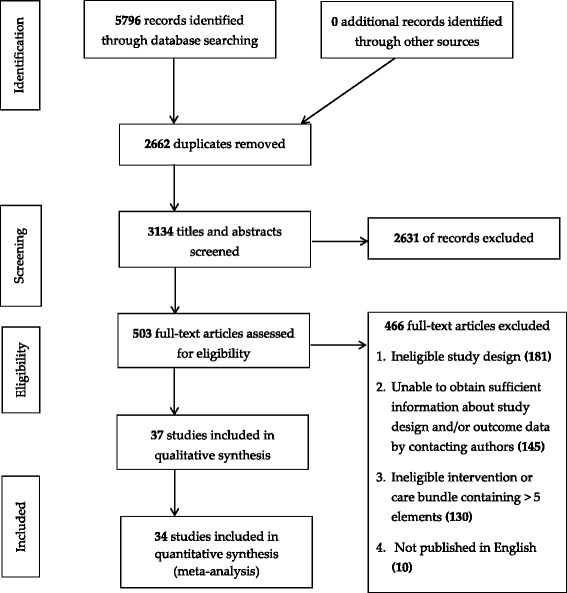
PRISMA flow diagram to identify eligible studies

The initial search generated 5796 records, and a total of 37 met the criteria for inclusion in the review (Fig. 1). The reasons for excluding records are stated in Fig. 1.

### Study characteristics

The characteristics of each study are presented in detail in Additional file 2. We identified four individually randomised trials [49–52], two cluster randomised trials [53, 54] and 31 controlled before-after studies [55–82]. All of the included studies reported on care bundles within inpatient settings. A variety of health conditions were targeted with ventilator-associated pneumonia being the most common [56, 59, 61, 66, 71, 74, 82]. Two studies reported the implementation of two bundles [53, 73] and one study reported on three care bundles [63]. Descriptions of the people delivering the care bundles were limited. The duration of the intervention varied from 3 months to 7.5 years (the median length of time was 31.5 months).

A variety of behaviour change techniques were used to facilitate the implementation and the potential success of the care bundles (see Additional file 2). However, no study reported a theoretical basis for choosing the various behaviour change techniques. ‘Feedback and monitoring’ was the most commonly reported behaviour change technique used to support the implementation of the care bundles (reported in 22 studies) (see Additional file 2). Eight studies reported using an implementation framework or psychological theory to inform the implementation of the care bundles [42, 48, 55, 63–66, 72].

#### Risk of bias

Summaries of the risk of bias assessments for the included randomised trials and non-randomised trials are presented in Additional file 3. The cluster-randomised trial [53], which aimed to improve the consistency of stroke care through the implementation of a care bundle, was assessed as being at low risk of bias. Three randomised trials and one cluster-randomised trial were at high risk of bias [50–52, 54], and one study was unclear for risk of bias due to poor reporting [49]. Two of the controlled before-after studies were assessed as having a low risk of bias [75, 83], eight were assessed to be at a moderate risk of bias [55, 58, 66, 69, 72, 76, 84], 15 were assessed to be at a serious risk of bias [56, 57, 59–65, 67, 68, 71, 74, 78, 85] and seven were assessed to be at a critical risk of bias [70, 73, 77, 80, 81].

#### Effects of care bundles

**Fig. 2:**
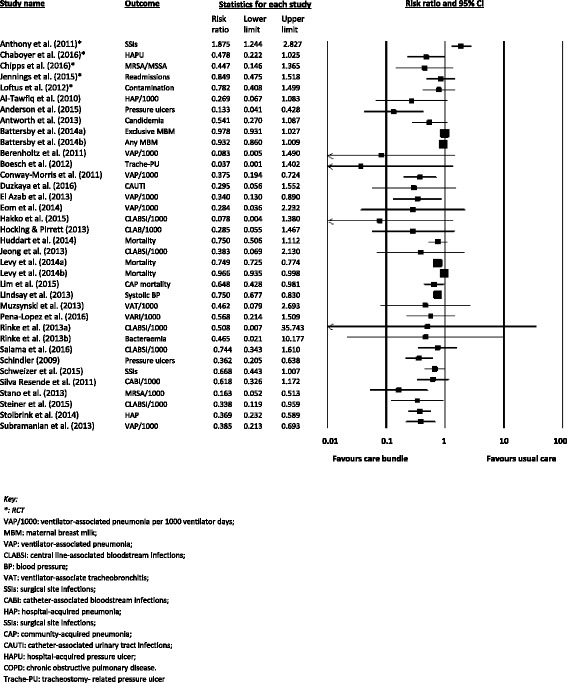
Effects of care bundles on patient outcomes. A forest plot of the risk ratios for each of the included studies

**Fig. 3:**
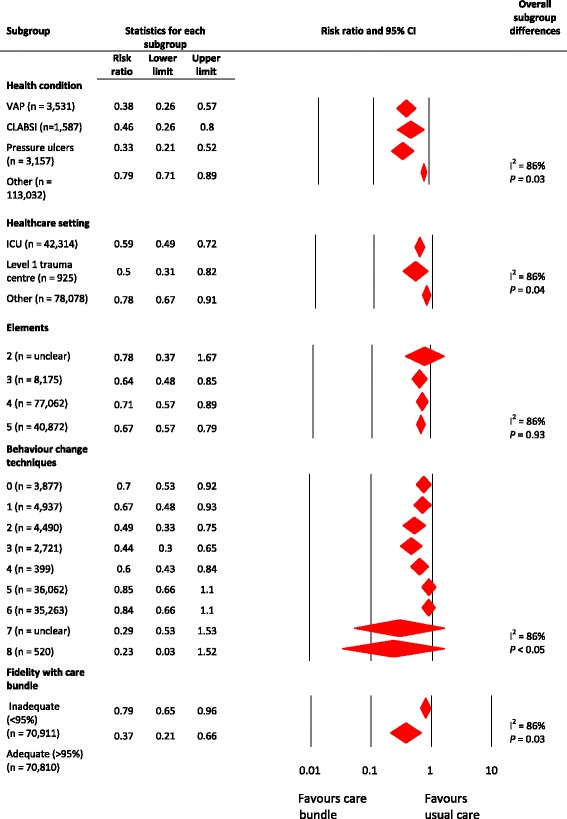
Subgroup analysis of included studies. An analysis of five subgroups including risk ratios and heterogeneity

There was a substantial variation in the effect of care bundles across the individual studies, ranging from a RR of 0.08 (care bundle decreased the risk of ventilator-associated pneumonia [74]) to a RR of 1.88 (care bundle increased the risk of surgical site infections [50]) (see Fig. 2) (Chi-squared = *P* < .1; *I* ^2^ = 86%). As a consequence of this heterogeneity, we did not pool all of the data into one analysis. Rather, we used subgroup analysis to explore cautiously whether specific methodological features and intervention aspects of the care bundles might impact on the relative effects (see Fig. 3). One randomised trial [53] and one controlled before-after study [72] did not report any patient outcomes, only fidelity with the care bundles, and it was not possible to re-analyse the findings from Smith [77] due to insufficient information. Thus, these three studies are not included within this section of the analysis. Due the limited number of studies, we could not conduct a meta-regression.

### Impact of study features on effect sizes

There were insufficient comparisons involving patient outcomes to enable a meta-regression of study features and the magnitude of the effects (Fig. 3). Below, we report on the findings of the subgroup analysis.

#### Study design

Given that observational studies are generally at a higher risk of bias than non-randomised studies, we compared the results of randomised and non-randomised studies. The pooled treatment effect for the randomised trials (*n* = 2049) was, on average, RR 0.97 [95% CI 0.71 to 1.34]. There were five randomised trials included in the analysis, and the findings are likely to be driven by one randomised trial which found an increase in the number of negative events and was stopped early [50]. The difference in the between subgroup effects for the randomised trials and controlled before-after studies was statistically significant. The controlled before-after studies generated a greater average treatment effect in favour of care bundles than the randomised trials (*n* = 119,178; RR = 0.66 [95% CI 0.59 to 0.75]). However, the quality of the evidence was very low quality (downgraded for risk of bias, inconsistency and indirectness).

#### Health condition

We grouped studies by their target health condition to assess impact (Fig. 3). The care bundles appeared to be potentially effective in all of the conditions we evaluated (including a heterogeneous ‘other’ group). The test for differences between subgroups was statistically significant (*P* < 0.001) suggesting that there were differences in effects between the subgroups. The studies assessing the effects of care bundles on the incidence of pressure ulcers, central line-associated bloodstream infections and ventilator-associated pneumonia may have the largest reductions in the risk of negative patient outcomes. A small reduction was observed for the heterogeneous ‘other’ category. However, we considered all these data to be of very low quality due to risk of bias, inconsistency and indirectness.

#### Healthcare setting

The data also suggested care bundles were potentially effective across all of the settings in which they were evaluated (Fig. 3). Care bundles may be more effective in trauma and ICUs compared with the heterogeneous ‘other’ group. However, this is very low quality evidence (downgraded for risk of bias, inconsistency and indirectness).

#### Care bundle elements of care

We assessed whether the number of elements of care within the care bundle impacted on patient outcomes (Fig. 3). Whilst all care bundles (regardless of the number of elements) reduced the risk of the negative patient outcomes, the test for differences between the subgroups was not statistically significant (*P* = 0.93). The RR was similar irrespective of the number of elements. For example, for three elements, the RR was 0.64 [95% CI 0.48 to 0.85] and for five elements, the risk ratio was 0.67 [95% CI 0.57 to 0.79]. However, the quality of evidence within this subgroup was very low (downgraded for risk of bias, inconsistency and indirectness).

#### Behaviour change techniques

The frequency with which the behaviour change techniques were delivered was often not reported nor were the levels of engagement with the behaviour change techniques. We assessed the impact of the number of behaviour change techniques on the effectiveness of care bundles. There were significant variations between the subgroups and the lowest risk for the negative patient outcomes was in the subgroup with ‘eight behaviour change techniques’ (RR = 0.23 [95% CI 0.03 to 1.52]) (Fig. 3). The apparent effect of care bundles appeared to reduce as the number of elements increased (the care bundles with five elements had an RR of 0.85 [95% CI 0.66 to 1.1]). However, we considered these data to be of very low quality due to risk of bias, inconsistency and indirectness.

#### Fidelity with the care bundle

Fidelity with the care bundle elements was regarded as adequate at 95% or above. As hypothesised, adequate fidelity (three studies) may be associated with a larger effect on patient outcomes (RR = 0.37 [95% CI 0.21 to 0.66]) when compared with inadequate fidelity (RR = 0.82 [95% CI 0.66 to 1.0]). However, the evidence was of very low quality which was downgraded for risk of bias, inconsistency and indirectness.

## Discussion

This systematic review was the first step towards gaining an extensive understanding of care bundles in general. We have identified a large, heterogeneous body of research which shows that care bundles may be an effective intervention for improving patient outcomes in acute settings (e.g. preventing ventilator-associated pneumonia in ICUs). However, the certainty of our conclusion is greatly tempered by the low or very low quality of the evidence (with most of the evidence coming from controlled before-after studies). We have shown that the care bundles evaluated using the non-randomised designs are more likely to report greater patient benefits. This is likely to be due, at least in part, to the biases in the study design and conduct. Unfortunately, the evidence from the randomised trials was uncertain (five studies with a total sample size of *N* = 2049).

Existing systematic reviews of care bundles are condition or setting-specific and suggest that care bundles may be effective in preventing and managing a range of conditions such as sepsis [28], central line-associated bloodstream infections [30] and chronic obstructive pulmonary disease [26]. Others focussed on hospital settings [36, 38, 86]. Across all of the existing reviews, the certainty of the evidence was deemed to be low and the high risk of bias in the included studies continues to be reported, limiting the certainty of the conclusions about the effectiveness of care bundles.

It was difficult to assess the effect of fidelity to the care bundles on patient outcomes. Thirteen studies reported levels of fidelity with the care bundle. Levels of adherence varied between the studies suggesting that the full implementation of the elements of care included in the care bundles was rare. This is an important issue as three studies demonstrated fewer occurrences of the negative events (central line-associated bloodstream infections [62], mortality [73] and surgical site infections [60]) when fidelity with the care bundle was high. However, within the analysis, we were generally working with uncertain data, and review findings must be considered in line with the observational nature of subgroup analysis. As noted previously, the quality of the evidence is very low and therefore, we are uncertain whether there was an underlying effect of care bundles that is independent of these study characteristics.

A systematic review of 47 non-randomised studies [29], reporting the strategies used to facilitate the implementation of care bundles employed on ICUs, found the most frequently used strategies were audit and feedback, education and reminders. Unfortunately, the findings were inconclusive as implementation fidelity was rarely reported and the certainty of the evidence was assessed as being low. Thus, it was not possible to determine the most effective strategies used to improve the uptake of the care bundles. These findings are similar to those reported in this review and in a review of 14 studies (five controlled trials, two interrupted time series studies, seven controlled before-after studies) evaluating the effectiveness of chronic obstructive pulmonary disease discharge care bundles [26]. The poor reporting of the implementation fidelity issues may restrict the utility and reproducibility of the systematic review findings [87]. Thus, clear reporting of intervention components and of implementation fidelity are essential to the complete interpretation of data about the effectiveness of behaviour change interventions.

The lack of theory in the development and implementation of the care bundles was evident throughout the systematic review. Eight studies reported using an implementation framework or a psychological theory to guide their implementation [53, 59, 66, 74–77, 83]. When encouraging healthcare workers to use evidence-based strategies, taking a theory-informed approach is recommended [19, 88]. However, often a pragmatic approach is taken, and this lack of explicit psychological theory during the design and implementation phases of the care bundle may impact on the effectiveness of such interventions [89–91].

Mechanisms of action are the theoretical constructs through which behaviour change techniques have their effect. Explicitly stating the potential mechanisms of action (e.g. restructuring the environment, training) can facilitate the generalisation of the care bundle findings to other healthcare settings. The most commonly used behaviour change techniques were ‘feedback and monitoring’ and ‘shaping knowledge’. This is in line with previous findings on implementation strategies [37, 92, 93]. However, the frequency of these behaviour change techniques was often not reported, and neither were the levels of engagement with the behaviour change techniques (e.g. attendance at training sessions), or the mechanisms of action. Thus, conclusions regarding the effectiveness of using the behaviour change techniques to facilitate a change in the behaviours of healthcare workers were not possible.

### Limitations

Our systematic review had some limitations. Firstly, we did not explore the strength of the evidence underpinning the care bundles. It is possible that the elements themselves have contributed to the heterogeneity, but it was not within the scope of the current review to assess the content of the care elements*.* Secondly, behaviour change techniques used in each study were coded retrospectively according to the Behaviour Change Technique Taxonomy Version 1 [22]. Thus, we are unsure whether these behaviour change techniques were intentionally used to increase the uptake of the care bundles.

Finally, our search terms were broad and the data are heterogeneous with high variability among health conditions, settings, care bundle elements and outcomes, thus the comparisons are limited. Existing systematic reviews have taken a more narrow, condition or setting-specific approach, so reducing the potential for drawing overall conclusions about the effects of care bundles. One of the aims of the systematic review was to evaluate the evidence of care bundles in general to assess the generalisability and consistency of the research findings across a wide range of study populations. As the review question was broad, we did not apply narrow inclusion criteria for the systematic review which is likely to have increased the number of eligible studies and allowed a more detailed exploration of heterogeneity as well as reducing the likelihood of type I error [94].

By ‘lumping’ studies together initially, a more detailed understanding of care bundles was possible through the subgroup analyses (specified a priori). The subgroup analysis assisted in strengthening the process as the advantages of lumping and splitting were combined [95]. Whilst the existing reviews provide information about the effectiveness of care bundles in highly specified situations, there is little understanding of their effects in general. Consequently, this systematic review was the first step towards identifying and addressing gaps in the care bundle literature. However, taking such a broad scope was problematic for two reasons. Firstly, it was likely to have increased the level of statistical heterogeneity. Secondly, it was difficult to balance the impact of the exploration with the clarity required for a meta-analysis. A cautious approach to interpreting the findings from the subgroup analysis is necessary as they are observational in nature [44] and therefore are at risk of bias through confounding by other study-level characteristics [96].

### Future research

This systematic review has highlighted interesting but very low quality data. The need for clear and unambiguous reporting has been highlighted during this review especially with regards to who is delivering the care bundle and the content of the implementation intervention. The TIDieR checklist for interventions [97] needs to be followed more rigorously.

## Conclusions

Very low quality evidence from controlled before-after studies (downgraded due to the risk of bias, inconsistencies and potential indirectness of outcomes) suggests that the implementation of care bundles may be an effective strategy to improve patient outcomes when compared with usual care. By contrast, the low-quality evidence from five randomised trials (downgraded due to the risk of bias, inconsistencies and potential indirectness of outcomes) is highly uncertain. Future research should focus on the explicit and transparent reporting of the implementation of the care bundle including issues relating to implementation fidelity such as the frequency with which the behaviour change techniques were used. The higher quality reporting of the research findings will enable stronger conclusions to be drawn about the effectiveness of care bundles.

## Additional files

Additional file 1: Search terms. Search strategy performed in each database. (DOCX 27 kb)

Additional file 2: Summary of included studies [98–108]. (DOCX 76 kb)

Additional file 3: Summaries of risk of bias. Review authors’ judgements about each risk of bias item presented as percentages across all included studies. (DOCX 17 kb)

## Abbreviations

CBAs: Controlled before-and-after studies
CI: Confidence interval
ICU: Intensive care unit
MD: Mean differences
RCTs: Randomised controlled trials
RR: Risk ratio
SMD: Standardised mean difference
