# Supplementary material for: The effects of care bundles on patient outcomes: a systematic review and meta-analysis
Source: Implement Sci. 2017 Nov 29;12:142. doi: 10.1186/s13012-017-0670-0 (PMC5707820; doi:10.1186/s13012-017-0670-0)
Supplement: Supplementary file 1 — Search terms. Search strategy performed in each database. (DOCX 27 kb) [file 13012_2017_670_MOESM1_ESM.docx]

**OVID Databases (inc. PsycInfo and EMBASE and Medline)**

1 Patient care bundle*.tw,fs,sh,mp.

2 exp Care bundle/

3 Care adj bundle*

4 exp Patient care bundle

5 Care adj checklist

6 Care checklist.mp,fs.

7 Prevention adj bundle

8 Prevention bundle.mp.

9 1 or 2 or 3 or 4 or 5 or 6 or 7 or 8

10 guidelines as topic/ or practice guidelines as topic/  
11 Guideline Adherence/
12 exp Critical Pathways/  
13 (guideline? not (guideline? adj2 author?)).ti,ab.  
14 ((pathway? or protocol? or algorithm?) adj2 (clinical or treatment? or diagnos$ or 15 management or infection? or infectious? or antibiotic?)).ti,ab.
16 critical pathway?.ti,ab.
17 guidance.ti,ab.  
18 (quality adj2 (improv$ or manag$ or care or healthcare)).ti,ab.  
19 (guideline? adj2 (impact or effect$)).ti,ab.

20 10 or 11 or 12 or 13 or 14 or 15 or 16 or 17 or 18 or 19

21 9 and 20

CENTRAL

1 Patient care bundle*

2 Patient care bundle* MeSH descriptor

3 Patient care bundle*:ti

4 Patient care bundle*:ab

5 Patient care bundle*:kw

6 Care NEXT bundle*

7 Care NEXT checklist

8 Care checklist:ti

9 Care checklist:ab

10 Care checklist:kw

11 or/1-10

12 Prevention NEXT bundle

13 Prevention bundle*

14 Prevention bundle*:ti

15 Prevention bundle:ab

16 Prevention bundle:kw

17 or/12-17

18 11 and 17

CINAHL:

1 Patient care bundle*MH

2 Patient care bundle*MH+

3 Patient care bundle*Wn

4 “Patient care bundle”

5 “care bundle”

6 care bundleMH+

7 Care checklistWn

8 Care checklistMH

9 Care checklistMH+

10 “Care checklist”

11 Prevention bundleWn

12 Prevention bundle*

13 Prevention bundle*MH

14 Prevention bundleMH+

15 “prevention bundle”

16 1 or 2 or 3 or 4 or 5 or 6 or 7 or 8 or 9 or 10 or 11 or 12 or 13 or 14 or 15

PROQUEST databases:

1 Patient care bundle*

2 Patient care bundle*P/n

3 “Patient care bundle”

4 “care bundle”

5 Care checklist

6 Care checklistP/n

7 “Care checklist”

8 Prevention bundle*

9 “Prevention bundle”

10 Prevention bundle/n

11 1 or 2 or 3 or 4 or 5 or 6 or 7 or 8 or 9 or 10

British nursing index:

(Patient OR patient care team OR caregivers OR medical staff OR nurs* OR nurse practitioners OR nurse clinicians OR nurses community health OR nurses public health OR nurse’s aides OR health personnel OR humans) AND (patient care bundle OR patient safety OR patient care management OR guideline OR complex intervention OR quality improvement OR guideline adherence OR practice guideline OR evidence-based practice OR nursing process OR evidence-based nursing OR evidence-based medicine OR evidence-based emergency medicine OR nursing OR care package OR care checklist OR prevention bundle OR care intervention OR care pathway) AND (control groups OR normal care) AND (intervention studies OR controlled before-after stud* OR interrupted time series analysis OR historically controlled stud* OR non-randomised controlled trial as topic OR control groups) AND (mortality OR patient care OR treatment outcome OR nurs* practice patterns OR quality improvement OR patient outcome assessment OR treatment outcome* OR effectiveness OR outcome assessment OR quality of healthcare OR comparative effectiveness research OR program evaluation OR patient harm OR compliance OR adherence OR incidence)

Search term key: exp = explode subject heading; adj = adjacency searching, / = subject heading, .tw. = textword, $ = truncation; .mp. = searches title, abstract, full text, caption text, ti = title, ab = abstract, mh = subject heading
