# Supplementary material for: The effects of care bundles on patient outcomes: a systematic review and meta-analysis
Source: Implement Sci. 2017 Nov 29;12:142. doi: 10.1186/s13012-017-0670-0 (PMC5707820; doi:10.1186/s13012-017-0670-0)
Supplement: Supplementary file 2 — Summary of included studies [98–108]. (DOCX 76 kb) [file 13012_2017_670_MOESM2_ESM.docx]

| **Study** | **Design** | **Setting** | **Participants** | **Care bundle and implementation strategies** | **Outcomes** | **Study author reported findings** |
| --- | --- | --- | --- | --- | --- | --- |
| Al-Tawfiq et al. [56] | Controlled before-after study. | Conducted in Saudi Arabia in an adult ICU. | Sample size, and inclusion and exclusion criteria unclear.  Those involved in delivering the care bundle: multidisciplinary team (including infection control professional, critical care nurses, respiratory therapist, intensivist). | Intervention:  Ventilator-associated pneumonia (VAP) care bundle consisting of 5 elements (head of bed elevation; a daily ‘sedation vacation’; readiness to wean assessment; peptic ulcer disease prophylaxis; deep vein thrombosis prophylaxis).  Comparator: usual care.  Behaviour change techniques:  Instruction on how to perform behaviour; self-monitoring of behaviour.  Study duration: 2 years. | Primary outcome:  (1) cases of VAP/1,000 ventilator days);  Secondary outcome:  (1) fidelity with the care bundle. | Cases of VAP/1,000 ventilator days reduced from 9.3 to 2.1.  Fidelity with the care bundle improved from 20% to 82%. |
| Anderson et al. [57] | Controlled before-after study. | Conducted in USA on a level 1 trauma centre. | 327 adult patients.    Inclusion criteria:  Patients admitted from emergency department/ transferred from inpatient units to ICUs.  Exclusion criteria:  Patients less than 18 years of age, presence of a pressure ulcer, previous study enrolment, ICU length of stay less than 24 hours.  Those delivering the care bundle: wound, ostomy, and continence (WOC) nurses. | Intervention (n = 146):  Universal pressure ulcer prevention bundle care bundle consisting of 5 elements (skin emollients, assessment of skin head to toe, floating heels off bed, early identification of sources of pressure, using pressure redistribution surfaces, repositioning).  Comparator (n = 181): standard care.  Behaviour change techniques:  Instruction on how to perform behaviour; feedback on outcome(s) of behaviour; problem solving; identification of self as role model.  Study duration: 12 months. | Primary outcome:  (1) incidence of unit-acquired pressure ulcers.  Secondary outcome:  (1) fidelity with the care bundle. | Unit-acquired pressure ulcer incidence rates reduced from 15.5% (28 patients [pressure ulcers were stage 2]) to 2.1% (3 patients [pressure ulcers were stage 1, 2, and 3]).  In the pre-intervention phase, 7 patients developed multiple stage 2 pressure ulcers (2-5 per patient); whereas in the post-intervention phase, of the patients who developed pressure ulcers, it was one pressure ulcer.  Fidelity with the practices included in the care bundle did not differ between the pre- and post-intervention phases  (*P* = 0.123). Although, fidelity with repositioning and elevation of heels improved (*P* = 0.015 and *P* < 0.001, respectively). |
| Anthony et al. [50] | Randomised trial, randomisation by block method (computer generated 50 subjects per block generated by principle investigator), randomisation sequence concealed prior to assignment, allocation 1 to 1 between intervention and control arms. | Undertaken in USA. | 210 adult patients.  Those delivering the care bundle:  Unclear.  Inclusion criteria:  Patients having elective transabdominal colorectal procedures (undergoing laparoscopic and open procedures, and diverting and bypassing procedures).  Exclusion criteria:  Patients undergoing emergency operations, transrectal procedures, procedures involving only small bowel or appendix. | Intervention (n = 106):  Care bundle made up of 5 elements (omission of mechanical bowel preparation, use of preoperative and intraoperative warming designed to maintain normothermia, maintenance of increased concentration of inspired oxygen during and immediately after surgery, reduction of intravenous fluids during the operation, use of wounds edge protection). Also received anti-biotics prior to surgery.  Comparator (n = 104):  Received anti-biotics prior to surgery and current practices.  Behaviour change techniques:  Identification of self as role model; practical social support; monitoring of behaviours by others without feedback; instruction on how to perform behaviour.  Study duration: 35 months. | Primary outcomes:  (1) surgical site infections (overall infection rate at 30 days after surgery);  (2) time to identification.  Secondary outcome:  (1) fidelity with the care bundle. | 45% of the patients in the intervention group developed a surgical site infection compared with 24% in the control group. Those receiving the care bundle were at significantly more risk of developing a surgical site infection when compared with the control group  (*P* = 0.003).  The median time to identification of infection was 9 days and there were no significant differences between the 2 groups (*P* = 0.71).  Fidelity with the care bundle: 84% patients received all 5 elements, 99% received at least 4 elements. |
| Antworth et al. [58] | Controlled before-after study. | Conducted in a level 1 trauma centre in USA. | 78 patients.  Inclusion criteria: candidemia >1 positive blood culture; adult and paediatric patients.  Exclusion criteria: Patients who died during the study period.  Those delivering care bundle: infectious diseases physicians, pharmacists, physicians. | Intervention (n = 41):  Comprehensive care bundle consisting of 5 elements (selection of appropriate antifungal therapy based on culture and susceptibility results, removal of intravascular catheters, repeat blood cultures at least every 48 hours until negative, appropriate duration of antifungal treatment, ophthalmologic examination to evaluate candida endophthalmitis).  Behaviour change techniques:  Instruction on how to perform behaviour.  Comparator (n = 37):  Usual care.  Study duration: 18 months. | Primary outcome:  (1) fidelity with the care bundle.  Secondary outcomes:  (1) length of stay;  (2) time to clearance of blood cultures;  (3) persistent blood cultures within 72 hours;  (4) recurrent candidemia within 4 weeks. | Fidelity with the care bundle was higher in the post intervention group (78%) compared with the pre intervention group (40.5%; *P* = 0.002).  No differences in the secondary outcomes from pre intervention to post intervention were found:  Length of stay (21 days and 20 days respectively;  *P* = 0.918).  Time to clearance of blood cultures (3 days for both groups;  *P* = 0.61).  Persistent blood cultures > 72 hours (40.5% and 22% respectively;  *P* = 0.126).  Recurrent candidemia within 4 weeks (5.4% and 4.9% respectively; *P* = 0.916). |
| Battersby et al. [75] | Controlled before-after study. | Conducted in England, UK in neonatal units. | 33,172 neonatal patients.  Inclusion criteria:  ≤32 +6 weeks gestation admitted to neonatal unit.  Exclusion criteria:  None stated.  Those delivering the care bundle:  Multidisciplinary team (including: midwives, lactation specialists, maternity support workers, neonatologists, neonatal nurses, neonatal support workers, nursery nurses, breastfeeding specialists, dieticians, speech and language therapists). | Intervention (n = 3680):  Care bundle consists of 4 elements (promotion of early milk expression, ongoing support for expressing and breast feeding up to discharge, standardised enteral feeding practice, aseptic non-touch technique for milk preparation).  Comparator (n = 29,429): Standard care.  Implementation based on Plan-do-Study-Act framework proposed by Bevan et al. [98]  Behaviour change techniques:  Instruction on ow to perform behaviour; self-monitoring of behaviour.    Study duration: 4 years. | Primary outcomes:  (1) monthly percentage of infants receiving exclusive maternal breast milk at discharge;  (2) monthly percentage of infants receiving any maternal breast milk at discharge;  (3) monthly percentage of care days where any maternal breast milk was received.  Secondary outcome:  (1) fidelity with care bundle;  (2) completeness of discharge feeding data. | Percentage of infants receiving exclusive maternal breast milk at discharge increased faster in the intervention group  (*P* = 0.01).  The number of infants receiving any maternal breast milk at discharge increased faster in the intervention group  (*P* = 0.001).    The percentage of care days where infants received maternal breast milk significantly increased (*P* = 0.03).  Fidelity with the care bundle increased from approximately 12% to 80%.  Completeness of discharge feeding data improved from 85% to 99%, and handwritten medical notes improved from 57% to > 80%. |
| Berenholtz et al. [74] | Controlled before-after study. | Conducted in USA in 112 ICUs across 72 hospitals. | Sample size and inclusion/exclusion criteria unclear.  Those delivering care bundle: unclear. | Intervention (80 ICUs):  Ventilator care bundle consisting of 5 elements (semi-recumbent positioning to decrease risk of VAP, stress ulcer prophylaxis to reduce deep vein thrombosis, adjustment of sedation until patient can follow commands, daily assessment of readiness to extubate).  Comparator (81 ICUs):  Usual care.  Implementation based on model for organisational change Sinuff et al. [93].  Behaviour change techniques:  Instruction on how to perform behaviour; monitoring of behaviour by others without feedback.  Study duration: 36 months. | Primary outcome:  (1) VAP cases per 1,000 ventilator days.  Secondary outcome:  (1) fidelity with the care bundle. | Decrease in VAP cases per 1000 ventilator days from 5.5 cases (pre-intervention) to 0 cases (post-intervention; *P* < .001).  Fidelity with the care bundle elements increased from 32% (pre-intervention) to 84% (post-intervention;  *P* < 0.001). |
| Boesch et al. [76] | Controlled before-after study. | Undertaken in USA in an 18 bed ventilator unit. | 843 paediatric patients.  Those delivering the care bundle:  Multidisciplinary team.  Inclusion criteria:  Tracheostomy-dependent patients.  Exclusion criteria:  None stated. | Intervention (n = unclear):  Tracheostomy-related pressure ulcer prevention bundle made up of three elements (pressure risk and skin assessment, moisture-free device interface, pressure-free device interface).  Comparator (n = unclear):  No details provided.  Implementation based on Plan-Do-Study-Act cycles Langley et al. [99].  Behaviour change techniques:  Instruction on how to perform behaviour.  Study duration: 30 months. | Primary outcome:  (1) tracheostomy-related pressure ulcers (new pressure ulcer developed after patient admitted/transferred to unit and in direct contact with tracheostomy).  Secondary outcomes:  (1) tracheostomy days;  (2) fidelity with the care bundle. | Significant reduction in the number of new pressure ulcers per month in tracheostomy patients from 8.1% to 0.3%  (*P* < 0.05).  The number of tracheostomy days also decreased from 12.5% to 0.2%. |
| Chaboyer et al. [54] | Cluster-randomised trial. Random number generating software was used to randomise hospitals (clusters) within strata, with random 1:1 block allocation of hospitals to intervention or control group. | Conducted in Australia in 8 tertiary referral hospitals with acute medical, surgical and rehabilitative services. | 1598 adult patients.  Inclusion criteria: Patients aged ≥18 years; had an expected hospital length of stay ≥48 hours; at risk of pressure ulcer as measured by limited mobility (i.e. requiring physical or mechanical assistance to reposition or ambulate); able to read English and provide informed consent. A screening log was kept to identify patients who did and who did not meet the inclusion criteria. Limited mobility rather than a pressure ulcer risk assessment score was used by recruiters to screen for eligibility.  Exclusion criteria:  Patients were excluded if they were: admitted to the hospital for >36 h prior to recruitment; admitted to day surgery, critical care, emergency, maternity, paediatrics, mental health or dialysis; previous trial participants; palliative, or receiving end of life care.  Those delivering the bundle: nurses, dieticians. | Intervention (n = 799):  Pressure ulcer prevention care bundle consisting of three elements (keep moving, look after your skin, eat a healthy diet).  Comparator (n = 799):  Standard pressure ulcer prevention care.  The care bundle was informed by the concept of patient participation in care (Sahlsten et al., 2008), pressure ulcer prevention clinical practice guidelines [100], and five systematic reviews on pressure ulcer prevention [101-105]. No theoretical basis for implementation. Behaviour change techniques used included instruction on how to perform behaviour, prompts/cues, monitoring of behaviour by others without feedback.  Study duration:  11 months. | Primary outcome:  (1) incidence of hospital-acquired pressure ulcers/1000 patient follow up days.  Secondary outcomes:  (1) severity of hospital-acquired pressure ulcers;  (2) patient participation in pressure ulcer prevention. | Hospital-acquired pressure ulcer incidence reduced from 20.1/ 1000 person-days (control group) to 9.6/1000 person-days (intervention)  (*P* > 0.05).  There was no significant difference between intervention and control groups in the severity of new pressure ulcers or in patient participation in pressure ulcer prevention.  No adverse events or harms were reported. |
| Chipps et al. [52] | Randomised trial.  Upon extubation, randomisation occurred using a  computer-generated table of random numbers. | Conducted in USA in a large academic medical centre (including ICUs and cardiac surgical unit). | 69 adult patients.  Inclusion criteria:  Patients who were mechanically ventilated for at least 48 hours and were being considered for ventilator liberation (criteria PEEP < 8 and FiO2 < 50%) or had been recently extubated. In addition, subjects were required to have a minimum of three teeth and be able to provide informed consent, either directly or through a legally authorised representative.  Exclusion criteria:  allergy to products or components of the oral care protocol or a history of oral or facial surgery or trauma in the 3 months prior to enrolment. Bleeding disorders (identified as an INR *>* 3.5 or platelet count *<* 20,000), planned hospital discharge within 48 hours, current diagnosis of mucositis, current chemotherapy or radiation therapy, and presence of tracheostomy; or when family or the attending physician was not in favour of continued medical treatment.  Those delivering the care bundle: clinical nurse specialists from the ICU areas. | Intervention (n = 30):  Oral care protocol consisting of 5 elements (tooth brushing; tongue scraping; flossing; mouth rinsing; lip care).  Comparator (n = 39):  Usual care (Patients who can manage their secretions are provided with hospital-purchased dental products, and oral care is provided per hospital policy including tooth brushing, mouth rinsing, and lip balm. Nursing assistance is provided as needed. Patients who have difficulty managing their secretions post-extubation receive routine oral care by the nursing staff using a toothbrush attached to suction. In this setting, routine documentation of oral hygiene does not include the type of product used).  Behaviour change techniques: instruction on how to perform the behaviour; demonstration of the behaviour.    Study duration: 36 months. | Primary outcomes:  (1) R-THROAT score;  (2) incidence of MRSA and MSSA.  Secondary outcomes:  (1) symptom burden using the Edmonton Symptom Assessment System;  (2) patient satisfaction with the oral care products and oral hygiene program. | R-THROAT scores decreased significantly in the intervention group demonstrating a large effect size (d = 0.79) and significant improvements when compared with the control group (*P* = 0.04).  The incidence of MRSA and MSSA did not differ between the intervention and control groups  (*P* = 0.45).  Patients receiving the intervention reported less drowsiness than those receiving usual care (*P* < 0.05), there were no differences in other aspects of symptom burden (*P* > 0.05).  Subjects in the intervention group reported higher staff attention to oral care (*P* = 0.05). Overall, patients in the intervention group rated their toothbrushes, toothpaste, mouthwash, and lip balm products higher than the usual care group. |
| Conway-Morris et al. [66] | Controlled before-after study. | Conducted in Scotland, UK in an 18-bed medical/surgical ICU. | 1961 patients.  Inclusion criteria: patients admitted to ICU for 48 hours or more during study period.  Exclusion criteria: unclear.  Those delivering the care bundle: unclear. | Intervention (n = 501):  Ventilator-associated pneumonia prevention bundle consisting of 5 elements (daily sedation hold, daily trial of ventilator weaning for suitable patients, head-up position, chlorhexidine mouth care, subglottic secretion drainage using specialised endotracheal tubes).  Comparator (n = 1460): Standard infection control precautions.  Implementation was based on Plan-Do-Study-Act cycles.  Behaviour change techniques:  Goal setting (behaviour); action planning; self-monitoring of behaviour, monitoring of behaviours by others with feedback; feedback on outcomes of behaviour; practical social support; emotional social support.  Study duration: 4.5 years. | Primary outcomes:  (1) VAP cases per 1,000 ventilator days;  (2) anti-biotic use;  (3) rates of MRSA.  Secondary outcomes:  (1) duration of mechanical ventilation;  (2) ICU length of stay;  (3) ICU mortality;  (4) fidelity with the care bundle. | VAP cases/1000 ventilator days reduced from 32 (pre-intervention) to 12 (post-intervention;  *P* < 0.001). This included a reduction in the rates of both clinical VAP rates from 15% to 9% (*P* < 0.001) and microbiologically confirmed VAP rates from 9% to 4%  (*P* = 0.002).  Antibiotic use did not change (*P* = 0.2).  MRSA incidence decreased from 10% (pre-intervention) to 3.6% (post-intervention;  *P* = 0.001).  The duration of mechanical ventilation did not change (*P* = 0.17).  No differences were found in length of stay (*P* = 0.5).  Mortality rates reduced from 25% to 20% (*P* = 0.03).  Fidelity with all bundle elements was 70%. |
| Duzkaya et al. [78] | Controlled before-after study. | Conducted in Turkey in a paediatric ICU. | 750 child patients.  Inclusion criteria:  Patients aged between 1 month and 18 years who stayed in the paediatric ICU for over 48 hours and had no symptoms of urinary infection during this time.  Exclusion criteria:  Patients staying less than 48 hours, patients with positive urine culture before admission or within 48 hours of stay on paediatric ICU.  Those delivering the care bundle: 13 nurses. | Intervention (n = 390):  Catheter-associated urinary tract infections (CAUTI) prevention bundle consisting of five elements (perform hand washing before and after contact with each  patient’s catheter and drainage system, and use sterile gloves when inserting the catheter;  for female patients, separate the labia minora and cleanse the vulval area with sterile water before catheterisation;  for male patients, clean the urethral meatus with sterile water and ensure the foreskin is retracted if present; use a new silicone catheter per insertion with a closed sterile drainage system using a sterile technique; evaluate daily catheter requirement).  Comparator (n = 360):  Usual care.  Behaviour change techniques employed included instruction on how to perform the behaviour, demonstration of the behaviour, feedback on outcomes of behaviour.  Study duration: 24 months. | Primary outcome:  (1) CAUTI/1000 catheter days.  Secondary outcomes:  (1) length of stay;  (2) mean mechanical ventilation;  (3) mean catheterisation time;  (4) mean catheterisation time in patients with CAUTI. | CAUTI reduced significantly from 6.1/1000 catheter days to 1.8/1000 catheter days (*P* = 0.001). CAUTI rate was 6% at baseline compared with 1.5% post-intervention.  Length of stay reduced from 14 days to 13 days following the introduction of the care bundle.  There were no differences in the mean mechanical ventilation.  The mean catheterisation time reduced from 10 days to 8 days, and the mean catheterisation time in patients with CAUTI reduced from 20 days to 17 days. |
| El Azab et al. [59] | Controlled before-after study. | Undertaken in Saudi Arabia in an ICU. | 992 adult patients.  Inclusion criteria: not stated.  Exclusion criteria: not stated.  Those involved in delivering the care bundle: multidisciplinary team (ICU nurses, respiratory therapist, clinical pharmacist, infection control coordinator, quality management department). | Intervention (n = 800):  Ventilator care bundle consisting of 4 elements (head of bed 30-45 ͦ ; daily sedation vacation; peptic ulcer disease prophylaxis; deep vein thrombosis prophylaxis). An additional care element not stated as part of the care bundle was regular oral care with chlorhexidine (every 8 hours).  Comparator (n = 192):  Usual care.  Implementation based on the Theory of Planned behaviour as described by O’Keefe-McCarthy et al. [106].  Behaviour change techniques: monitoring of behaviours by others with feedback; self-monitoring of behaviour; instruction on how to perform behaviour.  Study duration: 2 years. | Primary outcomes:  (1) VAP (cases per 1000 ventilator days);  (2) fidelity with the care bundle.  Secondary outcomes:  (1) mortality;  (2) length of stay. | VAP rate reduced by 65.4% (from 16.2 to 5.5 patient/1000 ventilator day).  Fidelity with the care bundle reached 100%  Mortality rates reduced from 23.4% to 19.1% (*P* = 0.024).  Length of stay reduced from 9.7 days to 6.5 days (*P* < 0.001). |
| Eom et al. [61] | Controlled before-after study. | Undertaken in Korea in adult ICUs in 6 hospitals. | Sample size, and inclusion and exclusion criteria were not stated.  Those delivering the care bundle  (N = 324):  Doctors (n = 23), nurses (318). | Intervention:  Ventilator-associated pneumonia care bundle consisting of 4 elements (head of bed elevation check 4hourly; peptic ulcer disease prophylaxis daily; deep vein thrombosis daily; oral decontamination with chlorhexidine 0.12% 8 hourly) with an optional fifth element (continuous aspiration of subglottic secretions).  Comparator:  Usual care.  Behaviour change techniques:  Instruction on how to perform behaviour; self-monitoring of behaviour; monitoring of behaviours by others with feedback; practical social support; identification of self as role model.  Study duration: 12 months. | Primary outcome:  (1) (VAP/1000 ventilator days.  Secondary outcome:  (1) fidelity with the care bundle. | Incidences of VAP reduced from 4.1 (57 cases) to 1.16 (7 cases).  Overall fidelity with the care bundle increased from 41.1% to 71.8%. Fidelity with each element increased, except for peptic ulcer disease prophylaxis which decreased by 2%. |
| Hakko et al. [62] | Controlled before-after study. | Conducted in Turkey in a medical/surgical ICU. | Total sample size is unclear.  Inclusion criteria:  Patients admitted to ICU requiring central venous catheter.  Exclusion criteria: unclear.  Those involved in delivering the care bundle: head physician of the ICU, infectious disease physician, nurse manager of the unit, infection control nurse, physicians, nurses, technicians. | Intervention (n = 725):  Central line care bundle consisting of 4 elements (removal of all lines placed in the emergency room/in another hospital within 24 hours; use of aseptic technique, hand hygiene, and maximum barrier precautions; use of a dedicated lumen for total parenteral nutrition; total parenteral nutrition infusion sets changed in 24 hours). Had an addition 5 infection control precautions which were not part of the care bundle but were also implemented.  Comparator (n = unclear).  Behaviour change techniques:  Instruction on how to perform behaviour; self-monitoring of behaviour; monitoring of behaviours by others with feedback; feedback on outcomes of behaviour; prompts/cues; practical social support.  Study duration: 4 years. | Primary outcome:  (1) Central line-associated bloodstream infection (CLABSI) rates/1,000 catheter days.  Secondary outcome:  (1) Fidelity with the care bundle. | CLABSI/1,000 catheter days reduced from 22.9 to 0 following the introduction of the care bundle.  Fidelity with the care bundle was maintained at 100% in the post-intervention phase.  There was a strong negative correlation between fidelity with the care bundle and CLABSI rates  (*P* < 0.001). |
| Hocking & Pirrett [63] | Controlled before-after study. | Undertaken in New Zealand in a critical care complex which includes an ICU and high-dependency beds. | Sample size, patient characteristics, those delivering the care bundle, inclusion and exclusion criteria are unclear. | Intervention:  Depending on their clinical status patients received:  Institute for Healthcare Improvement central line insertion care bundle with 5 elements (hand hygiene and use of chlorhexidine 2% and alcohol to cleanse the skin prior to inserting central line, using subclavian vein as preferred site, full barrier precautions [hat, mask, sterile gown, gloves], full body drape, sterile technique whilst inserting central line and applying the dressing).  Maintenance care bundle consisting of 3 elements (infusing intravenous nutrition via a dedicated lumen, daily checking of the central line site for inflammation, cleaning of all ports with 2% chlorhexidine and 70% alcohol prior to accessing the central line).  A high risk care bundle consisting of 2 elements (chlorhexidine impregnated dressings and/or antibiotic  impregnated central lines) was given to those with: burns, neutropenia, prescribed immune-suppressants or central lines that had been rewired or inserted in other hospitals or during emergencies).  Comparator: usual care.  Behaviour change techniques:  Instruction on how to perform behaviour; monitoring of behaviours by others with feedback; feedback on outcomes of behaviour; practical social support; identification of self as role model.  Study duration: 3.5 years. | Primary outcomes:  (1) number of central line days;  (2) monthly number of central line associated bacteraemia per 1000 central line days;  (3) fidelity with the care bundle (all-or-none). | The number of central line days did not significantly differ following the introduction of the bundles (*P* = 0.14); but there were significant differences between line days for the pre and each of the post bundle periods. There were fewer line days in the post insertion bundle period (*P* = 0.01) and high risk bundle period (*P* = 0.18), but there was an increase in the number of central line days in the maintenance bundle period (*P* = 0.02).  Reduction in the mean central line associated bacteraemia per 1000 line days from 6.43 to 1.83 cases (*P* < 0.001).  Fidelity with the care bundle increased with the elements of the insertion bundles from 36% to 81%, as well as the maintenance bundle from 76% to 80%. |
| Huddart et al. [83] | Controlled before-after study. | Undertaken in England, UK (4 UK hospitals). | 726 adult patients.  Those delivering the care bundle:  Unclear  Inclusion criteria:  Age over 18 years; Expedited, urgent or emergency abdominal surgery via a midline upper or lower abdominal incision including: patients for whom the planned procedure was laparoscopic, but subsequently converted to open surgery, laparoscopic surgery with a simultaneous procedure via an abdominal incision (laparoscopically assisted open surgery or vice versa), patients requiring simultaneous general surgical thoracotomy, all emergency laparotomies irrespective of the root cause; for some patients this will be the first presentation of the abdominal pathology, others may be experiencing complications of earlier elective or urgent surgery; expedited, urgent or emergency major abdominal laparoscopic surgery, but excluding appendectomy or cholecystectomy.  Exclusion criteria:  Appendectomy of any type as the sole surgical procedure; cholecystectomy of any type as the sole surgical procedure; gynaecological laparoscopy or laparotomy of any type unless the primary pathology is proven to be general surgical; pancreatectomy of any type; surgery related to organ transplantation; surgery relating to sclerosing peritonitis; emergency laparotomy for vascular surgery; laparotomy or laparoscopy following trauma or penetrating injuries to the abdomen (e.g., blunt injury, gunshot or stabbing). | Intervention (n = 427):  Received emergency laparotomy pathway  quality improvement care bundle with 5 elements (initial assessment with early warning scores, early antibiotics,  interval between decision and operation less than 6 h, goal-directed fluid therapy, and postoperative intensive care).  Comparator (n = 299):  Usual care.  Implementation informed by Plan-do-Study-Act framework Langley et al. [107].  Behaviour change techniques:  None reported.  Study duration: 8 months. | Primary outcomes:  (1) number of lives saved;  (2) 30 day mortality rate;  (3) hospital mortality. | The number of lives saved per 100 patients increased in the post-intervention group (12.44%) compared with the pre-intervention group (6.47%; *P* < .001).  Overall risk of death decreased from 15.6% to 9.6% (*P* = 0.002).  The risk of 30-day mortality reduced from 14% in the pre-intervention group to 10.5% in the post-intervention group  (*P* = 0.152). When mortality rates were adjusted for the person’s risk of morbidity and mortality, this decrease became statically significant  (*P* = 0.003).  Hospital mortality decreased from  17⋅4% to 10⋅1%  (*P <*0⋅001). |
| Jennings et al. [49] | Single-centre randomised trial; randomised using a computer generated list to allocate a 1:1 ratio stratified by age and sex. | Undertaken in USA. | 172 adult patients.  Those delivering the care bundle:  Unclear.  Inclusion criteria:  Diagnosis of chronic obstructive pulmonary disease (COPD) with presence of acute exacerbation.  > 40 years old.  Current / ex-smoker of at least 20 pack-years.  Exclusion criteria:  Medical history of asthma, interstitial lung disease, bronchiectasis, presence of airway hardware, lung cancer, any other cancer expected to impact life expectancy, < 1 year active chemotherapy / radiation, active substance abuse, neuromuscular disorders affecting respiratory system, language barriers, living in nursing home, ICU stay during admission, significant delirium / dementia. | Intervention (n = 93):  Received care bundle with 5 elements (tobacco and smoke exposure, gastroesophageal reflux disease, anxiety/depression screening, COPD education, communication within 48 hours of being discharged). Also received additional care (systemic steroids, antibiotics, inhaler therapy at the teams discretion).  Comparator (n = 79):  Standard care.  Behaviour change techniques:  None reported.  Study duration: 3 years | Primary outcomes:  (1) readmission to hospital (within 30 days of discharge);  (2) emergency department visits for acute exacerbations of COPD in the 30 days following discharge.  Secondary outcome:  (1) Time to readmission. | Readmission rates to hospital were lower in the intervention group (19.4%) compared to the control group (22.8%), but there was not a significant difference (*P* > 0.05).  The risk of emergency department visits did not significantly differ between the 2 groups (*P* > 0.05).  Time to readmission was similar between groups (*P* > 0.05).  *Note:* This study was stopped earlier than anticipated due to lack of effect. |
| Jeong et al. [64] | Controlled before-after study. | Undertaken in South Korea in ICU department of a university-affiliated hospital. | 541 patients (388 adult, 153 children).  Inclusion criteria:  Patients admitted to ICU during study dates who had undergone central venous catheter (CVC) insertion.  Exclusion criteria:  Unclear.  Those delivering the care bundle: Unclear. | Intervention (309 adults, 139 children):  Central line bundle consisting of 5 elements (hand hygiene, maximum barrier precautions, chlorhexidine skin antisepsis, insertion site).  Comparator (79 adults, 114 children):  Usual care.  Behaviour change techniques:  Instruction on how to perform behaviour; demonstration of the behaviour.  Study duration: 33 months. | Primary outcomes:  (1) CLABSI incidence rates (number of central line infections/1,000 central venous catheter days);  (2) length of time until CLABSI occurrence.  Secondary outcome:  (1) fidelity with the care bundle. | CLABSI reduced from 4.7 cases/1,000 central venous catheter days to 1.8 cases (*P* = 0.076) in the adult population. CLABSI rates reduced in the paediatric population from 3.7 cases to 0 cases (*P* = 0.014).  The mean length of time until central line-associated bloodstream infection occurrence increased from 11.3 days (pre-intervention) to 13.4 days (post-intervention; *P* = .477).  Fidelity with the care bundle in adult populations increased from 0% to 37.1%  (*P* < .001). Increases in fidelity with maximum barrier precautions increased from 31.0% to 83.7%  (*P* < .001), and chlorhexidine skin antisepsis from 0.0% to 40.0% (*P* < .001) were observed. No differences in fidelity with hand hygiene were observed  (*P* = 0.317; and a decrease in fidelity with the use of alcohol and povidone-iodine from 100% to 43.2%  (*P* < .001), and the selection of the femoral vein as the insertion site from 6% to 2.7%, (*P* = 0.118).  Fidelity with the care bundle in the paediatric population increased from 0.8% to 20.1% (*P* < 0.001). Fidelity with the hand hygiene element did not increase (*P* > 0.05). Fidelity with maximum barrier precautions increased from 79.0% to 89.9%  (*P* < 0.01). Chlorhexidine skin antisepsis increased from 0.8% to 21.1%  (*P* < .001), as did the selection of the femoral vein as the insertion site increased after the intervention from 4.8% to 11.2%  (*P* > 0.05). The use of povidone-iodine alone or a mixture of alcohol and povidone-iodine was 77%. |
| Lawrence & Fulbrook [72] | Controlled before-after study. | Undertaken in Brisbane in two ICU departments in two metropolitan hospitals. | 315 adult patients.  Inclusion criteria:  Ventilated patients on ICU.  Exclusion criteria: Unclear.  Those delivering the bundle: intensive care nurses. | Intervention (n = 151):  Ventilator care bundle consisting of 4 elements (sedation hold, head of bed elevation, gastric ulcer prophylaxis, deep vein thrombosis prophylaxis) with feedback about ventilator care bundle levels of fidelity.  Comparator (n = 164):  The above care bundle without feedback.  Behaviour change techniques:  Instruction on how to perform behaviour; monitoring of behaviour by others without feedback; prompts/cues; practical social support; goal setting (outcome); adding objects to the environment; restructuring the environment  Study duration: 1 year. | Primary outcomes:  (1) fidelity with the care bundle (all-or-nothing and overall). | Fidelity with the care bundle increased in the experimental group, but this increase was non-significant (*P* > 0.05). |
| Levy et al. [73] | Controlled before-after study. | Conducted in hospitals in USA, Europe, and South America. | 29,470 patients.  Inclusion criteria:  Patients with a suspected site of infection; two or more systemic inflammatory response syndrome criteria; one or more organ dysfunction criteria.  Exclusion criteria:  None stated.  Those delivering care bundle: unclear. | Intervention (n = 20,086):  Depending on clinical status a patient would receive one of the following care bundles:  Surviving Sepsis Campaign sepsis management care bundle consisting of 4 elements (low-dose steroids administered for septic shock, drotrecogin alfa (activated) administered, glucose control maintained > lower limit of normal, but < 150 mg/dl (8.3 mmol/L), inspiratory plateau pressures maintained < 30 cm H2O for mechanically ventilated patients).  Surviving Sepsis Campaign sepsis resuscitation care bundle consisting of 5 elements (serum lactate measured, blood cultures obtained prior to antibiotic administration, broad-spectrum antibiotics administered within 3 hours for emergency department admissions and 1 hour for non-emergency department ICU admissions, in the event of hypotension and/or lactate > 4 mmol/L (36 mg/dl): a) Deliver an initial minimum of 20 ml/kg of crystalloid (or colloid equivalent); b) Apply vasopressors for hypotension not responding to initial fluid resuscitation to maintain mean arterial pressure (MAP) > 65 mm Hg, in the event of persistent hypotension despite fluid resuscitation (septic shock) and/or lactate > 4 mmol/L (36 mg/dl):  a) Achieve central venous pressure (CVP) of > 8 mm Hg;  b) Achieve central venous oxygen saturation (ScvO2) of  > 70%).  Comparison (n = 6,609):  Low adherence group (fidelity levels of < 15%).  Behaviour change techniques:  Instruction on how to perform behaviour; feedback on outcomes of behaviour; prompts/cues; practical social support.  Study duration: 7 years. | Primary outcomes:  (1) mortality rates;  (2) sepsis severity score.  Secondary outcome:  (1) fidelity with the care bundle. | Management care bundle:  Mortality rates were lower in the high adherence group (32.3%) compared with the low adherence group (33.8%; *P* < .05).  High levels of fidelity were found at 47.2% of sites. Sites which participated for at least 2 years had higher levels of fidelity (*P* = 0.01).  Resuscitation care bundle:  Rates of mortality were reduced in the high adherence group when compared with the low adherence group (29% and 38.6%, respectively;  *P* < .001).  Median severe sepsis scores were lower in the high adherence group (51) compared with the low adherence group (58; *P* <0 .001).  High levels of fidelity were found at 46.8% of sites. Sites which participated for at least 2 years had higher levels of fidelity (*P* < 0.001). |
| Lim et al. [79] | Controlled before-after study. | Conducted in UK hospitals. | 2,563 adult patients.  Inclusion criteria:  Aged over 16 years with symptoms suggestive of lower respiratory tract infection, radiologically confirmed community-acquired pneumonia (CAP) and treatment for CAP by the admitting clinical team.  Exclusion criteria:  Adults previously discharged from hospital within 10 days of admission.  Those delivering the care bundle:  Unclear. | Intervention (n = 196):  Community-acquired care bundle consisting of four elements including (chest X-ray obtained within 4 h of hospital admission in all adults with suspected CAP; oxygen assessment and prescription in keeping with BTS oxygen guideline; severity assessment, supported by the CURB-65 score; timely and targeted antibiotics given according to CAP severity within 4 h of admission)  Comparator (n = 1552):  Usual care.  Behaviour change techniques:  Unclear as implementation was reported to be at the discretion of each hospital.  Study duration:  13 months. | Primary outcomes:  (1) time to first chest x-ray <4h from admission;  (2) time to first antibiotic dose <4h from admission;  (3) adherence to BTS CAP Guidelines-recommended antibiotic choice;  (4) adherence to BTS CAP Guidelines-recommended antibiotic route of administration;  (5) assessment of oxygenation status.  Secondary outcomes  (1) 30-day inpatient (30-day IP) mortality; (2) length of hospital stay. | Time to chest X-ray ≤4h and oxygenation assessment were not associated with bundle delivery.  Time to first antibiotic ≤4 h was significantly better in the bundle group (adjusted OR 1.52, 95% CI 1.08 to 2.14, *P* = 0.016).  Guideline adherence according to antibiotic route and type did not differ significantly between the bundle and no bundle groups. Antibiotic route: 44% and 37% adherence, respectively (OR 1.34, 95% CI 0.95 to 1.90, *P* = 0.094). Antibiotic type: 29% and 25% adherence, respectively (OR 1.25, 95% CI 0.86 to 1.82, *P* = 0.247).  30-day IP mortality was significantly lower in the bundle group (9%) compared with the no bundle group (14%) (OR 0.59, 95% CI 0.37 to 0.95,  *P* = 0.030).  Length of stay was longer in the bundle group (median days: 6) compared with the no bundle group (median days: 5). |
| Lindsay et al. [65] | Controlled before-after study. | Conducted in USA in internal and family medicine clinics. | 4,111 adult patients.  Inclusion criteria:  New and existing diabetes patients with hypertension.    Exclusion criteria: Unclear.  Those delivering the care bundle: Physicians, nurse  Practitioners, physician assistants, licensed practical  nursing teams. | Intervention (n = unknown):  Hypertension in diseases care bundle consisting of 3 elements (standardised blood pressure process; order set of medications; a patient-identified behavioural goal).  Comparator (n = unknown):  Usual care.  Behaviour change techniques:  Instruction on how to perform behaviour.  Study duration: 34 weeks. | Primary outcomes:  (1) fidelity with the care bundle;  (2) percentage of change in patients achieving a blood pressure less than 130/80.  Secondary outcome:  (1) stakeholder satisfaction with care processes. | Fidelity with the care bundle unclear.  Overall, the number of patients with blood pressure > 130/80 reduced from 36.1% (baseline) to 26.3% (those who received the care bundle; *P* < 0.001).  Overall satisfaction levels did not change; but patients reported to have become more engaged with their care following the introduction of the care bundle (*P* = 0.007). |
| Loftus et al. [51] | Single blinded randomised trial, randomisation occurred via a computer generated list. | Undertaken in USA in a tertiary and level 1 trauma centre. | Adult patients, sample size unclear (572 operating rooms).  Those delivering the care bundle:  Unclear.  Inclusion criteria  Unclear.  Exclusion criteria  Unclear. | Intervention:  Received care bundle with 2 elements (hibiscrub, DOCit).  Comparator:  Received sterile set of IV tubing and open lumen stopcock set, 24 inch with 3-gang 4-way stopcocks, and T-connector.  Behaviour change techniques:  Instruction on how to perform behaviour; monitoring of behaviours by others with feedback; feedback on outcomes of behaviour; prompts/cues; demonstration of the behaviour; adding objects to the environment; identification of self as role model.  Study duration: 3 months. | Primary outcome:  (1) case-end bacterial contamination of ≥ 1 internal lumens of the patient IV stopcock (primary stopcock lumen samples).  Secondary outcome:  (1) incidences of 30 day postoperative infections (effluent samples). | The incidence of bacterial contamination of the primary stopcock was reduced when compared with standard caps (OR 0.79, 95% CI 0.63–0.98, *P* = 0.034).  No difference in the incidence of 30 day postoperative infections (*P* = 0.640) |
| Muszynski et al. [67] | Controlled before-after study. | Undertaken in USA in paediatric ICU. | 725 paediatric patients.  Inclusion criteria:  Patients admitted to ICU.  Exclusion criteria:  Patients admitted to cardiothoracic ICU, patients with tracheostomy and chronic need for mechanical ventilation.  Those delivering the care bundle: Physicians, nurses, respiratory therapists. | Intervention (n = 387):  Paediatric ventilator-associated pneumonia care bundle consisting of 5 elements (elevate head of bed at least 30 ͦ; oral care with chlorhexidine rinse at least every 4 hours; extubate as soon as possible; suction oral secretions before the endotracheal tubes (ETT) using separate catheter for oral secretions and use closed suctioning systems for ETT suctioning; perform hand hygiene between patient contacts).  Comparator (n = 338):  Usual care.  Behaviour change techniques:  Instruction on how to perform behaviour; feedback on outcomes of behaviour; prompts/cues.  Study duration: 33 months. | Primary outcomes:  (1) cases of ventilator-associated tracheobronchitis/  1,000 ventilator days);  (2) cases of VAP.  Secondary outcome:  (1) fidelity with the care bundle. | Cases of ventilator-associated tracheobronchitis/  1,000 ventilator days reduced from 3.9 to 1.8 (*P* = 0.04).  There was one case of VAP in the pre-intervention phase and none in the post-intervention phase.  Fidelity with the care bundle was unclear. |
| Pena-Lopez et al. [82] | Controlled before-after. | Undertaken in Denmark in a 16-bed medical–surgical paediatric intensive care unit. | 312 children.  Inclusion criteria:  All children admitted to the paediatric ICU who had received invasive mechanical ventilation (MV) for 48 h or longer were included.  Exclusion criteria: None.  Those delivering care bundle: unclear. | Intervention (n = 108):  Ventilator care bundle consisting of 5 elements (Elevation of the patient’s head from the bed to at least 30˚; a structured oral care protocol, including oral care with chlorhexidine solution 0.12% every 6 h and tooth brushing with a standard toothpaste every 12 hours; use of cuffed endotracheal tubes when not contraindicated; maintenance of tracheal tube/tracheostomy cuff pressure between 20 and 30 cmH2O; circuit changes only if the circuit becomes soiled or damaged).  Comparator (n = 96):  Usual care.  Behaviour change techniques included shaping knowledge and feedback on outcomes of behaviour.  Study duration: 23 months | Primary outcomes:  (1) ventilator-associated respiratory infection rate (VARI) including pneumonia/1000 ventilator days and ventilator-associated tracheobronchitis/1000 ventilator days;  (2) median time to development of a VARI from onset of mechanical ventilation.  Secondary outcome:  (1) Paediatric ICU mortality;  (2) ICU staff knowledge of evidence-based guidelines for the prevention of VAP. | Overall, VARI reduced from 11.03/1000 ventilator days to 6.27/1000 ventilator days. VAP reduced from 4.14/1000 ventilator days to 1.05/1000 ventilator days and VAT reduced from 6.89/1000 ventilator days to 5.23/1000 ventilator days  (*P* > 0.05).  The median time to the development of VAT from the onset of MV or previous VARI increased from 5.5 to 48 days (*P* < 0.05) but not VAP.  PICU mortality fell from 28.4% to 16.6% (RR 0.58, 95% CI 0.35–1.00).  Staff knowledge was not reported. |
| Power et al. [53] | Cluster-randomised trial, stratified-randomisation approach; hospitals stratified by stroke performance. Computer-generated list used to randomly allocate 12 hospitals to intervention and 12 to control group. Not possible to blind patients due to nature of study. | Undertaken in England. | N = 6,592  Inclusion criteria (for participating sites):  Minimum of 10 inpatient stroke beds, agreement to participate by chief executive and consultant, dedicated MDT stroke team, availability of case notes for review.  Exclusion criteria:  None stated.  Those delivering the care bundle:  Unclear. | Intervention (n = 3,533):  Early hours care bundle consisting of 4 elements (brain imaging, delivery of aspirin/alternative antiplatelet, swallow screen, weight assessment).  Rehabilitation care bundle consisting of 5 elements (physiotherapy assessment, occupational therapy assessment, mood assessment, documented evidence of multidisciplinary team goals set for rehabilitation, 50% of patients’ hospital stay on stroke unit).  Comparator (n = 3,059):  Normal care.  Implementation based on Model for Improvement.  Behaviour change techniques:  None reported.  Study duration: 12months | Primary outcome:  (1) fidelity with the care bundles. | Fidelity with the early hours care bundle:  Increased in intervention from 19.6% at baseline to 42.3%. Also increased in control group from 24.3% at baseline to 37.5%. Significant difference between control and intervention group  (*P* < 0.05). Largest relative difference in administering aspirin (*P* < 0.05).  Fidelity with the rehabilitation care bundle: increased in intervention from 27.3% at baseline to 46.2%. Increased in control group from 21.9% at baseline to 33.2%. Significant difference between control and intervention (*P* < 0.05). Use of mood assessment and rehabilitation goals significantly increased in intervention group when compared with control group  (*P* < 0.05; *P* < 0.001, respectively). |
| Rinke et al. [85] | Controlled before-after study. | Undertaken in USA in a children’s centre in a tertiary care hospital paediatric oncology group. | 520 unique paediatric patients.  Inclusion criteria: Unclear.  Exclusion criteria: Unclear.  Those delivering the care bundle: clinic staff, homecare agency nurses, patient families. | Intervention (n = 339):  Central line maintenance care bundle consisting of 3 elements (aseptic entry, aseptic central line component change, family assessment).  Comparator (n = 330):  Usual care.  Behaviour change techniques:  Instruction on how to perform behaviour; monitoring of behaviour by others without feedback.  Study duration: 4 years. | Primary outcomes:  (1) CLABSI per 1000 central line days;  (2) bacteraemia per 1000 central line days.    Secondary outcomes:  (1) fidelity with the care bundle. | Reduction central line-associated bloodstream infections (pre: 0.63/1000 central line days, post: 0.32/1000 central line days; *P* = 0.005).  Bacteraemia infections also decreased (pre: 1.27/1000 central line days, post: 0.59/1000 central line days; *P* < .001).  Fidelity:  Aseptic entries (100%); aseptic central line component change (85%); family assessment (81%). |
| Roquilly et al. [84] | Controlled before-after study. | Undertaken in France at two ICUs in a university hospital. | 499 adult patients with a brain injury (traumatic brain injury,  subarachnoid haemorrhage, stroke, or other).  Those delivering care bundle: physicians, residents, physiotherapists, nurses.  Inclusion criteria: Those requiring mechanical ventilation for more than 24 hours.  Exclusion criteria: early decision to withdraw  care (taken in the first 24 hours in ICU); death in the first 24 hours; or  inclusion in a randomised trial. | Intervention (n = 200):  Evidence-based weaning bundle consisting of 4 elements (lung protective ventilation, nutrition support, probabilistic antibiotherapy, systematic approach to extubation).  Comparator (n = 299):  Usual care.  Behaviour change techniques:  None reported.  Study duration: 4 years. | Primary outcomes:  (1) duration of mechanical ventilation;  (2) fidelity with the care bundle.  Secondary outcomes:  (1) percentage of patients with hospital-acquired pneumonia;  (2) ventilator-free days (at day 90);  (3) ICU-free days (at day 90);  (4) mortality. | Significant reduction in the mean duration of mechanical ventilation from 14.9 days in the pre-intervention group to 12.6 days in the post-intervention group (*P* = 0.02).  Overall fidelity with the care bundle significantly increased from 6% to 21% (*P* < 0.01).  The percentage of patients with hospital-acquired pneumonia significantly decreased from 575% at baseline to 47.5% in the experimental group (*P* = 0.03).  The mean number of ventilator-free days also significantly reduced from 54 to 64 days (*P* = 0.01).  The mean number of ICU-free days significantly decreased from 50 to 57 days (*P* = 0.01).  Mortality rates reduced in the experimental group but not significantly:  Mortality at day 90  (*P* = 0.51); mortality in ICU (*P* = 0.22). |
| Salama et al. [81] | Controlled before-after study. | Undertaken in an adult ICU in a teaching hospital in Kuwait. | 7161 patient days.  Inclusion criteria:  All patients who were admitted to the ICU with a stay longer than 48 hours during the study period and those fulfilling criteria for healthcare-associated infection.  Exclusion criteria:  None stated.  Those delivering the care bundle: unclear. | Intervention (n = unclear; 6474 patient days):  Central venous line bundle consisting of 5 elements (hand hygiene by inserter; maximal barrier precautions upon insertion by the physician inserting the catheter and sterile drape from head to toe to the patient; use of a 2% chlorohexidine gluconate (CHG) in 70% ethanol scrub for the insertion site; optimum catheter site selection; examination of the daily necessity of the central line).  Comparator (n = unclear; 7161 patient days):  Usual care.  Behaviour change techniques:  Monitoring of behaviours by others without feedback; instruction on how to perform behaviour.  Study duration: 26 months. | Primary outcome:  (1) CLABSI rate/1000 catheter days;  (2) central line days.  Secondary outcome:  (1) fidelity with care bundle. | CLABSI/1000 catheter days reduced from 14.9 at baseline to 11.08 during the intervention (*P* = 0.08).  Central line days reduced from 5367 at baseline to 5052 during the intervention (*P* = 0.34).  Fidelity with the care bundle ranged from 51% to 91%. |
| Schindler et al. [69] | Controlled before-after study. | Undertaken in USA in a paediatric intensive care unit. | 399 paediatric patients.  Inclusion criteria:  Children admitted to PICO (0-3m).  Exclusion criteria:  None stated.  Those delivering care bundle: unclear. | Intervention (N = 250):  SKIN bundle consisting of 4 elements (support surface, keep turning every 2 hours, improve moisture management/incontinence management, nutrition consultation).  Comparator (N = 149):  Usual care including pressure relieving surface.  Behaviour change techniques:  Instruction on how to perform behaviour; feedback on outcomes of behaviour; prompts/cues.  Study duration: 14 months. | Primary outcomes:  (1) pressure ulcers.  Secondary outcomes:  (1) severity of pressure ulcers;  (2) mortality;  (3) barriers/ facilitators to implementation. | Number of acquired pressure ulcers reduced from 28 patients in the pre-intervention phase to 17 in the post-intervention phase  (*P* < .001).  Pressure ulcer severity in intervention group: 17.4% stage 1, 60.9% stage 2, 4.3% stage 3, 17.4% not stages).  Mortality rates did not differ between the 2 groups (*P* = 0.21).  Barriers: competing demands on staff time.  Facilitators: having skin care products and support surfaces readily available. |
| Schweizer et al. [60] | Controlled before-after study. | Undertaken in 20 hospitals in USA. | 42,534 operations on 38,049 adult patients.  Inclusion criteria:  18 years or older; patients who have undergone hip/knee arthroplasty or primary cardiac operation through median sternotomy incision.  Exclusion criteria:  Patients with pre-existing infections at surgical site.  Hospitals who already implement all three elements.  Those delivering the bundle: unclear. | Intervention (n = 14,316):  Evidence-based bundle consisting of 3 elements (screening for s aureus, decolonising carriers, prescribing optimal perioperative anti-biotics).  Comparator (n = 28,218): standard practice.  Behaviour change techniques:  Instruction on how to perform behaviour; self-monitoring of behaviour; feedback on outcomes of behaviour; prompts/cues; practical social support.  Study duration: 48 months. | Primary outcome:  (1) complex (deep incisional/organ space) *S aureus surgical site infections (*SSIs)/10,000 operations.  Secondary outcomes:  (1) the patient’s postoperative  length of stay during the index admission;  (2) readmissions  for treatment of SSIs within 90 days post-operation;  (3) adverse events;  (4) fidelity with the care bundle. | Care bundle was associated with a reduction in complex *S aureus* SSIs from 36/10,000 operations to 21/10,000 operations.  The care bundle did not decrease the postoperative length of stay or readmission rates  (*P* > 0.05).  Adverse events: 4 patients reported mild skin irritation.  When fidelity with the care bundle was 100%, complex *S aureus* SSI rates decreased significantly  (*P* < 0.05), but the rates did not significantly decrease in the partially adherent/non-adherent groups  (*P* > 0.05). |
| Silva Resende et al.[68] | Controlled before-after study. | Conducted in Brazil in a neonatal ICU. | 251 neonatal patients.  Inclusion criteria: all neonates admitted to neonatal ICU requiring central venous catheter.  Exclusion criteria: unclear.    Those delivering the care bundle: unclear. | Intervention (n = 107):  Care bundle consisting of five elements (hand hygiene, using full-barrier precautions during the insertion of CVCs, cleaning the skin with chlorhexidine 0.2%, avoiding the femoral site if possible, removing unnecessary catheters).  Comparator (n = 144):  Usual care.  Behaviour change techniques:  Instruction on how to perform behaviour; monitoring of behaviours by others with feedback; feedback on outcomes of behaviour; prompts/cues; identification of self as role model.    Study duration: 14 months. | Primary outcomes:  (1) cases of catheter-associated bloodstream infection/1,000 central venous catheter days;  (2) number of bloodstream infections. | Catheter-associated bloodstream infections reduced from 24.1/1,000 central venous catheter days to 14.9 (*P* < 0.05).  Blood stream infections decreased from 32% to 19.6%  (*P* = 0.04). |
| Smith et al. [77] | Controlled before-after study. | Conducted in the USA in two medical-surgical ICUs in a large medical centre. | 447 adult patients.  Inclusion criteria:  Patients admitted to a 10-bed medical-surgical ICU who were delirium-negative.  Exclusion criteria:  ICU patients who were delirium-positive on admission, resided in the ICU for 4 months or longer.  Those delivering care bundle: nurses. | Intervention (n = unclear):  Delirium prevention bundle consisting of five elements (sedation cessation for patients receiving mechanical ventilation; pain management; sensory stimulation; early mobilisation; sleep promotion).  Comparator (n = unclear):  Usual care.  Implementation was based on Virginia Henderson’s Theory of Need [108]. Behaviour change technique: instruction on how to perform behaviour.  Study duration: 244 days | Primary outcomes:  (1) delirium status using the CAM-ICU;  (2) arousal using the RASS scale.  Secondary outcome:  (1) adherence with the bundle. | The risk for delirium reduced by 78% in those receiving the care bundle (OR 0.22; 95% CI, 0.08-0.56;  *P* = 0.001).  The median arousal score was lower in those receiving the care bundle (median arousal score: 1) compared with those in the usual care group (median arousal score: 2).  Compliance ranged from 80-88%. |
| Stano et al. [70] | Controlled before-after study. | Conducted in Italy on one ICU. | 1,008 patients.  Inclusion criteria: unclear.  Exclusion criteria: unclear.  Those delivering care bundle: unclear. | Intervention (n = 577):  MRSA antibiotic care bundle consisting of 4 elements (rapid screening on admission to ICU, contact precautions, single room or cohort isolation, and nasal decolonisation (mupirocin 2% ointment three times-a-day for five days).  Comparator (n = 431):  Usual care.  Behaviour change techniques:  Monitoring of behaviours by others with feedback.  Study duration: 45 months. | Primary outcomes:  (1) total MRSA infection rate per 1,000 admissions. | 20.8 patients per 1,000 admissions developed MRSA in the pre-intervention phase compared with 3.4 per 1,000 admissions in the post-intervention phase (*P* < 0.001). |
| Steiner et al. [80] | Controlled before-after study. | Undertaken in Austria in a neonatal ICU. | 526 very low birth weight neonatal patients.  Inclusion criteria:  Every admitted very low birth weight neonate.  Exclusion criteria:  None.  Those delivering care bundle: 26 medical consultants and residents. | Intervention (n = 358):  Simulation-based prevention bundle consisting of 3 elements (a simulation-based standardisation and education of a peripherally inserted central catheter (PICC) insertion technique; improvement of breast milk hygiene management by standardised hand hygiene trainings for mothers; compulsory standardised hygiene training for all staff members).  Comparator (n = 168):  Usual care.  Study duration: 3 years. | Primary outcome:  (1) CLABSI/1000 central line days. | CLABSI/1000 central line days reduced significantly from 13.9 at baseline to 9.5 during the following year and 4.7 in the year after (*P* < 0.001). The reduction remained significant when subgroups were separated by birth weight (<1000 g,  *P* = 0.0016; ≥1000 g,  *P* = 0.001). |
| Stolbrink et al. [55] | Controlled before-after study. | Undertaken in the UK in 1 respiratory and 1 elderly care medicine ward. | 1179 adult patients.  Those delivering the care bundle:  Unclear.  Inclusion criteria:  Patients admitted to the ward.  Exclusion criteria:  Patients admitted electively or whose primary reason was surgical. | Intervention (n = 678):  Received ‘Early mobility’ care bundle made up of 5 elements (enhance availability of walking aids, provision of occupational theory equipment to maximise independence, mobility charts, individual instructions and information above each bed, informing and encouraging staff to support movement).  Comparator (n = 501):  Received usual physiotherapy care only.  Behaviour change techniques:  Instruction on how to perform behaviour.  Study duration: 6 months. | Primary outcome:  (1) hospital-acquired pneumonia incidence.    Secondary outcomes:  (1) length of stay;  (2) rate of falls. | Early mobility care bundle was associated with hospital-acquired pneumonia incidence  (*P* = 0.001) and a shorter length of stay (*P* = 0.009).  There was no difference in the rate of falls between the pre- and post-intervention groups  (*P* > .05). |
| Subramanian et al. [71] | Controlled before-after study. | Conducted in Malaysia in an ICU department. | 266 patients.  Inclusion criteria: all patients admitted to ICU during study period.  Exclusion criteria: no pneumonia diagnosis, no head injury.  Those delivering the care bundle: 71 ICU nurses. | Intervention (n = 130):  Ventilator care bundle consisting of 5 elements (elevation of head of bed to 30 ͦ- 45 ͦ (constant assessment unless medically contraindicated), daily sedation hold, peptic ulcer disease prophylaxis using pantoprazole or ranitidine, deep vein thrombosis prophylaxis via administration of subcutaneous heparin or enoxaparin and application of anti-embolism stockings, daily oral care with the help of a suction toothbrush and chlorhexidine gluconate 0.05%.  Comparator (n = 136):  Usual care.  Behaviour change techniques:  Instruction on how to perform behaviour; self-monitoring of behaviour; monitoring of behaviours by others with feedback; feedback on outcomes of behaviour; prompts/cues; practical social support; emotional social support; problem solving; action planning.  Study duration: 4 months. | Primary outcomes:  (1) nurses’ knowledge;  (2) fidelity with the care bundle.  Secondary outcome:  (1) VAP incidence per 1,000 ventilator days. | Nurses’ knowledge of VAP and the care bundle increased  (*P* < 0.001).  Fidelity with the care bundle elements increased (*P* < 0.001).  VAP incidence reduced from 39.01 (22 cases pre-intervention) to 15.11 (7 cases post-intervention;  *P* < 0.001). |
