# Supplementary material for: The effects of care bundles on patient outcomes: a systematic review and meta-analysis
Source: Implement Sci. 2017 Nov 29;12:142. doi: 10.1186/s13012-017-0670-0 (PMC5707820; doi:10.1186/s13012-017-0670-0)
Supplement: Supplementary file 3 — Summaries of risk of bias. Review authors’ judgements about each risk of bias item presented as percentages across all included studies. (DOCX 17 kb) [file 13012_2017_670_MOESM3_ESM.docx]

Figure S1*.* Risk of bias of Included Randomised and Cluster-Randomised Controlled Trials (n = 6).

Figure S2. Risk of bias of Included Non-Randomised Studies (n = 31).
